# Application of YOLO-v7 and YOLO-v8 Transfer Learning Models in Breast Lesion Classification and Diagnosis

**DOI:** 10.2174/0115734056422406251128144702

**Published:** 2026-02-02

**Authors:** Kaiting Jiang, Yuegui Wang, Haiquan He, Hong Chen, Haolin Shen

**Affiliations:** 1 Department of Ultrasound, Zhangzhou Affiliated Hospital of Fujian Medical University, Zhangzhou 363005, China; 2 Clinical Medical College, Fujian Medical University, Fuzhou 350004, China

**Keywords:** Breast Lesion, Deep learning, Breast ultrasound images, Transfer learning, YOLO-v7, YOLO-v8

## Abstract

**Background:**

Early detection of breast cancer and accurate assessment of lesions are key goals of imaging evaluation. Ultrasound is widely used, but its diagnostic performance is influenced by complex image features, noise, and operator experience. Reducing operator dependence and improving accuracy are critical clinical issues.

**Methods:**

In this retrospective study, 7,025 breast ultrasound images from our center were annotated based on pathology and split into training, validation, and internal test sets (8:1:1). The Dataset of Breast Ultrasound Images was used as the external test set. YOLO-v7 and YOLO-v8 models were trained through transfer learning after data augmentation and balancing the classes. Performance was compared on internal and external test sets and was evaluated against a reader study.

**Results:**

YOLO-v7 and YOLO-v8 reached optimal performance at epochs 294 and 135, respectively. YOLO-v7 slightly outperformed YOLO-v8 on the internal test set, while YOLO-v8 achieved higher accuracy, recall, specificity, precision, and F1 score on the external test set. Both models showed significantly higher accuracy, specificity, and precision than the senior radiologist, with YOLO-v8 achieving a significantly higher F1 score.

**Discussion:**

YOLO-v8 demonstrated better generalization due to its anchor-free mechanism and deeper architecture, while YOLO-v7 showed signs of overfitting. Both models outperformed the junior radiologist and approached or exceeded the diagnostic performance of the senior radiologist, indicating potential to assist less experienced readers.

**Conclusion:**

YOLO-v7 and YOLO-v8 effectively classified breast lesions. YOLO-v8 showed faster convergence and higher diagnostic efficiency, suggesting strong potential for clinical application.

## 1 INTRODUCTION

Breast cancer is the most commonly diagnosed cancer worldwide and the leading cause of cancer-related death in women[1, 2]. According tothe InternationalAgencyfor Research on Cancer (IARC), there were approximately 2.3 million new cases of breast cancer and approximately 666,000 deaths from breast cancer in 2022, both ranking in the top three for cancer incidence and mortality [3]. Early diagnosis and intervention of breast cancer can substantially improve the survival rate of patients, reduce the complexity of treatment, and improve prognosis. Therefore, early detection of breast cancer and accurate assessment of lesions are primary goals of various imaging examinations [4].

Ultrasound has the advantages of being relatively inexpensive, radiation-free, and well tolerated, making it one of the commonly used technologies for early detection of breast cancer [5-8]. Radiologists can assess lesion boundaries, morphology, and internal echo characteristics using real-time dynamic images [9]. However, the complex characteristics and noise of ultrasound images increase diagnostic difficulty and operator dependence. To address this, the American College of Radiology (ACR) developed the Breast Imaging-Reporting and Data System (BI-RADS), which standardizes terminology and reporting. The BI-RADS system uses unified terminology and evaluation criteria to help radiologists accurately describe imaging findings and provide valuable diagnostic references for clinicians. However, studies show that when using BI-RADS to classify breast lesions, junior residents demonstrate lower inter-observer consistency and diagnostic accuracy than senior radiologists [10]. In addition, radiologists may assign higher categories to difficult-to-characterize lesions to avoid missed diagnoses, resulting in high false-positive rates for ultrasound [11]. Against this background, reducing operator dependence and improving diagnostic accuracy remain key clinical needs.

Computer-aided diagnosis (CAD) has attracted increasing attention. CAD technology can improve the detection rate of lesions, enhance detection sensitivity, and improve lesion characterization to increase detection specificity [12]. In particular, deep learning can automatically extract and identify multi-level image features. By inputting image data for training, this feature extraction process does not require human intervention. It can analyze all levels of the image, from low-level edges and textures to high-level shapes and structures, making it more objective and accurate than manual reading [13, 14].

Recent developments in medical image analysis across different modalities further highlight the potential of deep learning. Dual-3DM3AD, for instance, employs a mixed Transformer architecture with triplet pre-processing for early multi-class Alzheimer’s diagnosis [15], while a hybrid FrCN–(U-NeT) technique has achieved strong performance in skin cancer segmentation and classification [16]. In the field of breast cancer imaging, XAI-RACapsNet integrates an explainable ROI segmentation module and a relevance-aware capsule network for mammography-based detection, demonstrating the value of combining interpretability with advanced feature extraction [17]. Collectively, these studies illustrate the broader trend toward transformer-based, hybrid, and explainable network designs that inform current CAD system development.

Parallel to these transformer-driven advances, real-time object detection frameworks have evolved rapidly to meet the clinical need for fast and accurate lesion localization. Among them, You Only Look Once (YOLO) remains a leading framework for real-time detection and classification, with automatic feature extraction that can improve consistency and accuracy. Since the release of YOLO, the framework has been continuously optimized. In July 2022, YOLO-v7 was released, introducing an efficient network architecture, optimizing the feature extraction and fusion process, and demonstrating higher object detection performance and training efficiency than previous versions [18]. YOLO-v8, released in January 2023, further improved YOLO-v7 by introducing an anchor-free mechanism and a more advanced feature extraction architecture to enhance detection accuracy. It also optimized the positive and negative sample selection strategy and the training algorithm [19].

Currently, YOLO has been applied in studies of diseases such as nasopharyngeal carcinoma, nervous system tumors, and cervical lymphoma, achieving promising diagnostic results [20-22]. Based on this, we hypothesize that applying YOLO to the detection of breast cancer in ultrasound images and assisting radiologists in diagnosis can further improve diagnostic accuracy, which has not yet been reported.

This study simultaneously applied YOLO-v7 and YOLO-v8 for transfer learning and compared their performance in the classification and diagnosis of suspicious breast lesions, evaluating their potential and effectiveness in early detection of breast cancer and providing a new technical approach to improve clinical diagnostic accuracy.

## 2 METHODS

This retrospective study was approved by the Research Ethics Committee of Zhangzhou Hospital affiliated to Fujian Medical University (No: 2022KYB202). All experiments were performed following relevant guidelines and regulations. The ethics committee of our hospital waived the requirement for written informed consent.

### 2.1 Clinical Data

A retrospective study was conducted on 1589 patients who were diagnosed with breast lesions and underwent pathological biopsy of lesions in our hospital from June 2022 to December 2022. According to the inclusion and exclusion criteria, 1511 eligible patients with 2215 lesions were included in the study. The patients' ages ranged from 15 to 96 years, and the lesion diameters ranged from 2 to 108 mm.

Inclusion criteria: (1) presence of breast lesion visible on ultrasound, (2) undergoing puncture and/or surgical resection and pathological diagnosis after ultrasound examination, and (3) availability of the most recent ultrasound examination, including 2D and color Doppler images.

Exclusion criteria: (1) missing ultrasound or pathological data, (2) undergoing interventional treatment such as surgery, radiotherapy, and chemotherapy before ultrasound examination, (3) pathological biopsy site not corresponding to the lesion site visible on ultrasound, and (4) pathological results indicating borderline tumor.

### 2.2 Instruments and Methods

Images were acquired at our center in routine practice using 19 different ultrasound machines from 7 manufacturers by 41 radiologists of varying seniority, introducing natural variability in technique and image quality.

### 2.3 Datasets

#### 2.3.1 Internal and External Datasets

A total of 7,025 ultrasound images of 2,215 lesions from our center were included as an internal dataset after removing highly redundant images. This dataset comprised 1,758 benign lesions with 5,166 images and 430 malignant lesions with 1,859 images.

The Dataset of Breast Ultrasound Images (BUSI) is one of the most commonly used open-access datasets in the field of breast ultrasound image detection, covering 780 breast ultrasound images across three categories: normal, benign lesions, and malignant lesions [23]. This study collected 647 benign and malignant lesion images from the BUSI dataset as an external test set. During verification, some images in the BUSI dataset were found to have the following issues: duplicate images (125 images), non-breast area images (43 images), images containing multi-classified lesions (17 images), and images containing biopsy needles (5 images) [24]. After excluding these images, 457 images of benign and malignant lesions were finally included. For multi-lesion images, only lesions with pathological results for which masks were provided in the dataset were selected for comparison of the models and radiologists’ diagnoses.

#### 2.3.2 Data Segmentation and Labeling

All images in the internal dataset were selected and annotated by a radiologist with 23 years of experience using LabelImg software. The original ultrasound and pathology reports were referred to during the annotation process to ensure that the locations of all annotated lesions and their benign or malignant classifications were consistent with pathological results. The annotated images were randomly divided into a training set, a validation set, and an internal test set in a ratio of 8:1:1. The training set was used to train the models and served as the primary data source for learning and identifying lesion characteristics. The validation set guided hyperparameter tuning to improve model generalization. The internal test set was used to evaluate the models’ performance on previously unseen images and to compare the effectiveness of different models. The results of the internal dataset division are shown in Table **1**. The external test set was used to compare the diagnostic performance of the models with that of the radiologists.

#### 2.3.3 Data Augmentation

To balance the dataset categories and enrich the data to increase the generalization ability of the models, data augmentation [25] was performed on the images in the training and validation sets according to the ratio of benign to malignant lesions. The benign images were augmented tenfold relative to the original dataset, including rotation (10°, 75°, 150° counterclockwise; 10°, 75° clockwise), contrast adjustments (coefficients of -0.5 and +1.0), and noise processing (Gaussian noise with a standard deviation of 75.00 and 5% salt-and-pepper noise). The malignant images were augmented twentyfold relative to the original dataset, including rotation (10°, 55°, 75°, 100°, 150° counterclockwise; 10°, 55°, 75°, 100°, 150° clockwise), contrast adjustments (coefficients of -0.5, -0.8, +0.5, +0.8, +1.0), and noise processing (Gaussian noise with a standard deviation of 50.00 and 75.00, and 3% and 5% salt-and-pepper noise). These transformations emulate common ultrasound acquisition variations, such as minor probe-angle changes, gain/contrast settings, and noise levels, thereby reducing majority-class bias and improving malignant lesion recall. The enhanced datasets are summarized in Table **2**.

### 2.4 Model Selection and Training

**Fig. (1):**
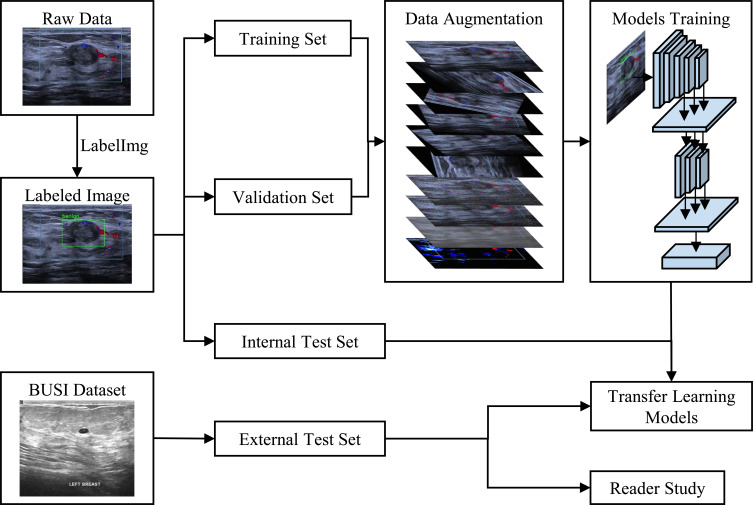
Study flow diagram.

Based on the enhanced dataset, the YOLO-v7 and YOLO-v8 models were trained for transfer learning on the same device. The source code and pre-trained weights of both models are publicly available on GitHub. Model training was implemented in Python 3.9.19 with PyTorch 1.11.0 (device configuration is shown in Table **3**, and the workflow is illustrated in Fig. **1**).

Key hyperparameters, including learning rate, batch size, input size, and intersection over union (IoU) and confidence thresholds, were initialized according to the official YOLO recommendations and further adjusted through stepwise tuning on the internal validation set to achieve stable convergence. During training, the models’ performance was evaluated by the mean average precision (mAP) on the validation set with an IoU threshold ranging from 0.50 to 0.95 (mAP50-95). The mAP was automatically calculated during the training process according to the following formula. Both models were set to train for 300 epochs, and an early stopping strategy was adopted. If a model failed to achieve better results for 50 consecutive epochs, training was terminated early to prevent overfitting.

**Table:** 

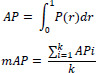

In addition, to assess computational feasibility, inference speed was measured on the external test set using the same hardware and software environment described above. Both YOLO-v7 and YOLO-v8 were tested with an input size of 640×640 and a batch size of 1, and the average per-image inference time, including preprocessing and postprocessing, was recorded.

### 2.5 Comparison of the Diagnostic Effects of the Models on the Internal Test Set

We used the internal test set to compare the diagnostic results of the trained YOLO-v7 and YOLO-v8 models. Given the high recall requirements in breast lesion diagnosis, the 50% IoU and 1% confidence thresholds were selected when using the models to diagnose lesions in test set images.

### 2.6 Models and Evaluation of Radiologists' Diagnostic Performance

To further verify the diagnostic performance of the models and compare it with that of radiologists, the two transfer learning models were tested on the external test set. Two radiologists, with 23 and 4 years of experience respectively, who had been trained in the BI-RADS 5th edition and had practical experience in breast lesion classification, participated in diagnosing the external dataset. During the radiologist evaluation, patient clinical information was concealed, and the radiologists classified the lesions solely based on features visible in the ultrasound images. Using BI-RADS 4A as the diagnostic cutoff, lesions classified as 4A and above were considered malignant, while those classified as 3 and below were considered benign.

A simulation of AI-assisted decision-making was performed to evaluate the potential clinical application of the YOLO-v8 model. Based on the model’s binary output, predefined upgrade and downgrade rules were applied to adjust the junior radiologist’s original BI-RADS category. If the radiologist assigned BI-RADS 2 or 3 and the AI predicted the lesion to be malignant, the category was upgraded to 4A. Conversely, if the radiologist assigned BI-RADS 4A or higher and the AI predicted the lesion to be benign, the category was downgraded to 3. After these adjustments, standard diagnostic metrics were recalculated.

### 2.7 Statistical Analysis

This study primarily used Python 3.9.19 for statistical analysis and visualization. In the internal test set, model performance was evaluated using the default YOLO output metrics, including mAP, recall, precision, and F1 score. To reflect the potential upper bound of model performance, the YOLO evaluation pipeline generated precision-recall (PR) curves and reported the operating point with the highest F1 score on the validation curve. The mAP value reported here corresponds to the highest value obtained when the IoU threshold was set to 0.50 (mAP50).

For the external BUSI dataset, which was designed to simulate clinical deployment and allow direct comparison with radiologists, a fixed decision threshold was applied. Accuracy, recall, specificity, precision, and F1 score were calculated directly from the confusion matrix to provide a practical estimate of generalizability. Accuracy was used to evaluate the correctness of overall predictions, while recall, specificity, precision, and F1 score were used to comprehensively assess diagnostic performance.

The formulas for these metrics are as follows. Among all lesions, those diagnosed as malignant and with actual malignant results were recorded as TP (True Positive), those diagnosed as non-malignant and with actual non-malignant results were recorded as TN (True Negative), those diagnosed as malignant but not actually malignant were recorded as FP (False Positive), and those diagnosed as non-malignant but actually malignant were recorded as FN (False Negative).

**Table:** 

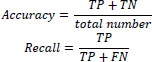

**Table:** 

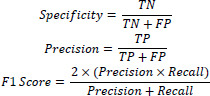

Pairwise case-level comparisons between each model and the senior radiologist used McNemar’s exact test for accuracy, sensitivity, and specificity. Differences in precision and F1 were assessed *via* 10,000-sample paired bootstrapping to estimate 95% CI. p values were adjusted with the Benjamini–Hochberg false discovery rate (FDR, q = 0.05). A bootstrap 95% CI excluding 0 indicated statistical significance.

## 3 RESULT

### 3.1 Model Training Results

Finally, the convergence speed of the YOLO-v7 model gradually stabilized about 55 epochs after the start of training and achieved the best results in the 294th epoch; the convergence speed of the YOLO-v8 model stabilized after about 30 epochs, achieved the best results in the 135th epoch, and triggered the early stopping mechanism after the 184th epoch to stop training. As shown in Fig. (**2**), all training and validation metrics demonstrated stable convergence without noticeable oscillation.

### 3.2 Comparison Results of Internal Model Testing

**Fig. (3):**
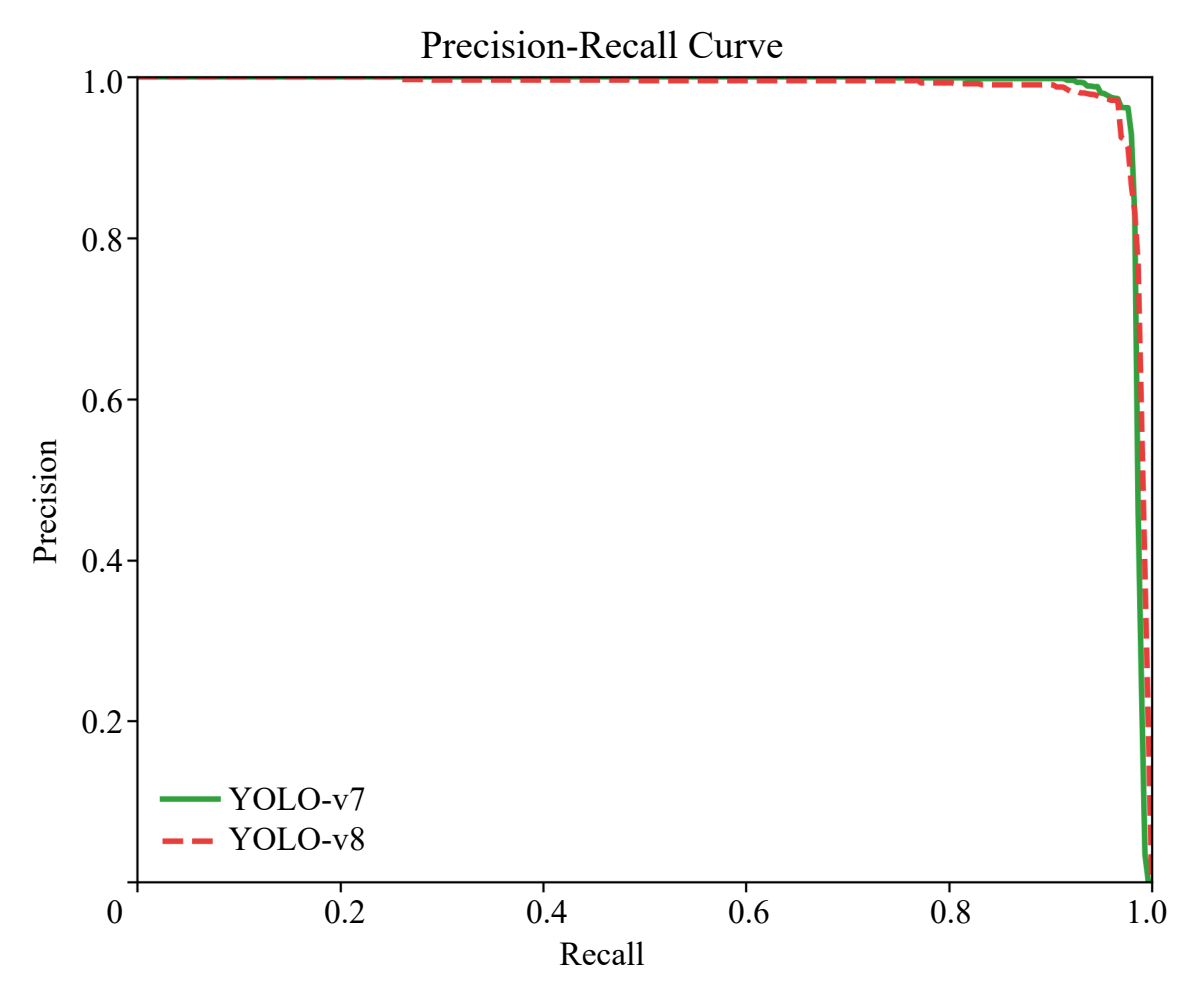
P-R curve comparison of YOLO-v7 and YOLO-v8 models in the internal test set.

We compared the diagnostic performance of the two models on the internal test set obtained from our center. On this set, the mAP50, recall, precision, and F1 score of the YOLO-v7 model were 0.983, 0.961, 0.980, and 0.970, respectively, while the corresponding metrics for the YOLO-v8 model were 0.981, 0.972, 0.959, and 0.965. The PR curve of model performance shows that YOLO-v7 was slightly superior to YOLO-v8 (Fig. **3**).

Inference speed was additionally evaluated on the internal test set to assess computational efficiency. The YOLO-v8 model achieved an average per-image inference time of 3.9 ms, corresponding to approximately 250 frames per second (FPS), whereas the YOLO-v7 model required 6.9 ms per image, or approximately 145 FPS.

### 3.3 Comparison of Diagnostic Results from Models and Radiologists on the External Test Set

**Fig. (5):**
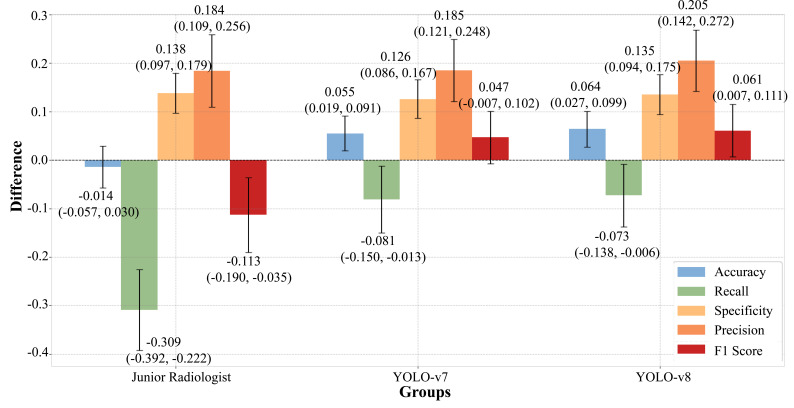
Diagnostic metrics differences between the junior radiologist and models compared to the senior radiologist on the external test set.

The two deep learning models were tested on an external test set. At the same time, senior and junior radiologists also interpreted images from the external test set according to the BI-RADS classification, and the diagnostic results are shown in Fig. (**4**). The YOLO-v8 model had the highest accuracy, precision, and F1 score, at 0.894, 0.924, and 0.830, respectively; the junior radiologist had the highest specificity, at 0.971; and the senior radiologist had the highest recall, at 0.821 (Table **4**). Bootstrap analyses using the senior radiologist as the reference confirmed these differences, showing significant precision gains for both YOLO-v8 (ΔPrecision = 0.142–0.272, 95% CI) and YOLO-v7 (ΔPrecision = 0.121–0.248, 95% CI). YOLO-v8 also achieved a significant improvement in F1 score (ΔF1 = 0.007–0.111, 95% CI), whereas junior radiologists demonstrated significantly lower sensitivity, specificity, and F1 scores (Fig. **5**). McNemar testing with FDR correction further supported these findings, indicating that YOLO-v8 significantly outperformed the senior radiologist in overall accuracy (p = 0.0021) and specificity (*p* < 0.001), with a trend toward higher sensitivity that did not reach significance (p = 0.0502). YOLO-v7 also showed significant improvements in accuracy (p = 0.0078) and specificity (*p* < 0.001), with a smaller gain in sensitivity (p = 0.0472). Fig. (**6A**-**C**) shows the diagnostic effect of the models on the images of the external test set.

### 3.4 Simulation of AI-assisted BI-RADS Adjustment

**Fig. (6):**
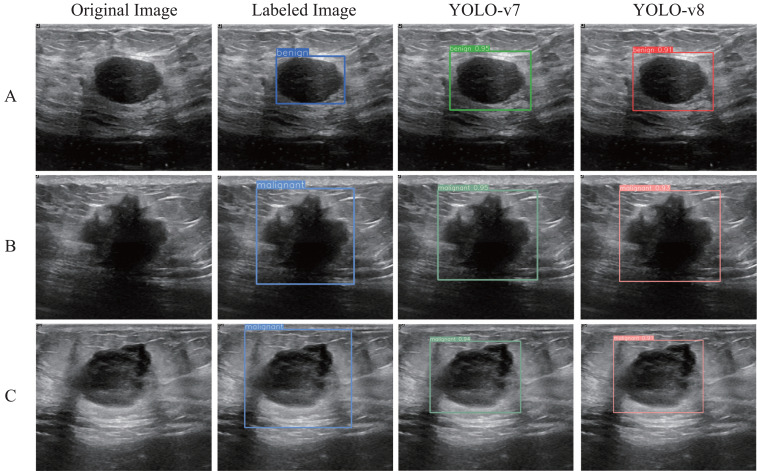
Examples of diagnostic performance of the two models on the external test set. **(A).** Pathology confirmed a benign lesion, with YOLO-v7 diagnosing it as benign at a confidence level of 0.95 and YOLO-v8 at 0.91. (**B).** Pathology confirmed a malignant lesion, with both models diagnosing it as malignant at confidence levels of 0.95 and 0.93. (**C).** Pathology confirmed a malignant lesion. Junior radiologist misclassified it as benign, while senior radiologist and both models diagnosed it as malignant, with confidence levels of 0.94 and 0.91.

**Fig. (7):**
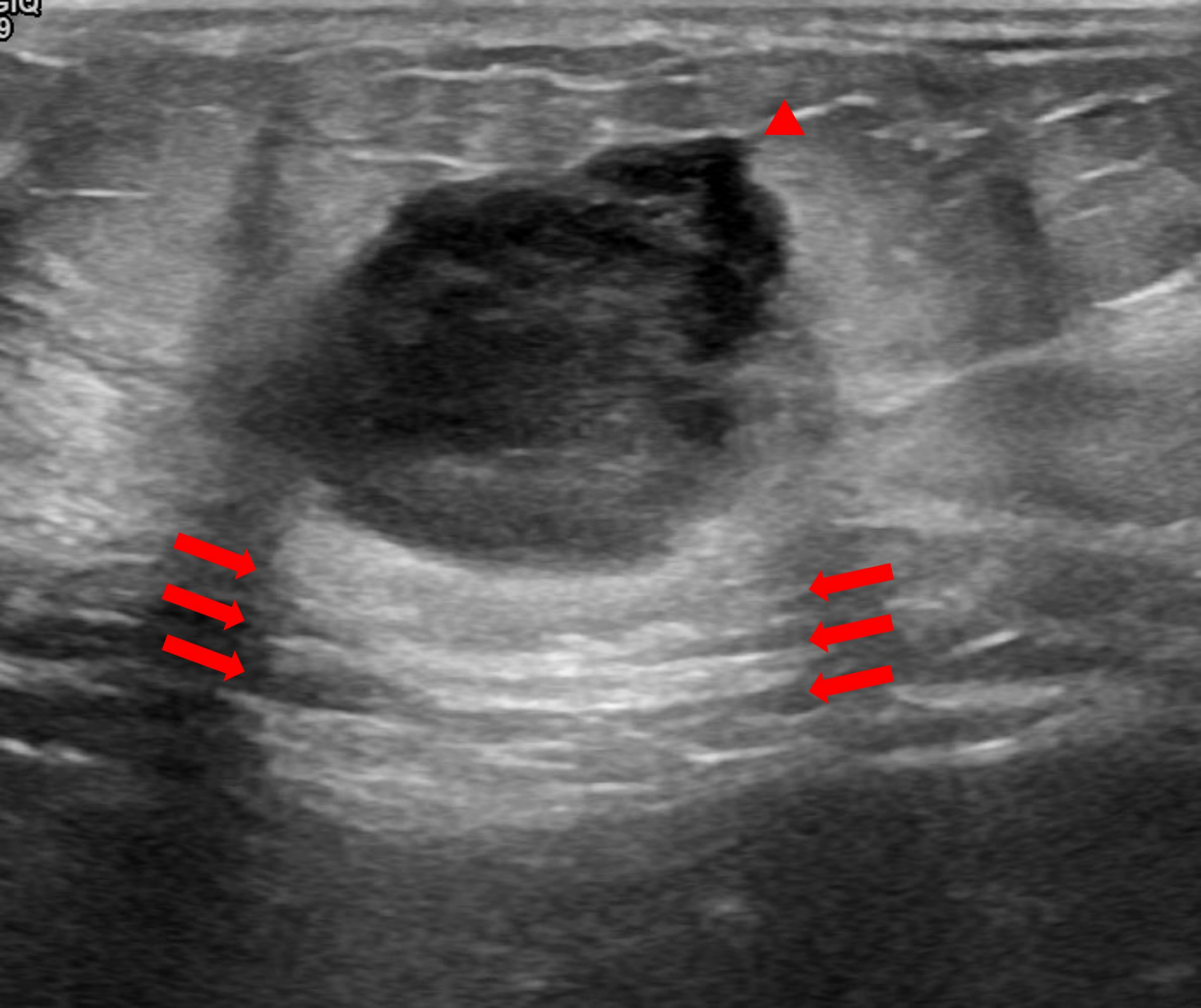
Ultrasound features of breast lesions. The ultrasound features of the lesion are as follows: (1) hypoechoic, (2) irregular shape, (3) heterogeneous internal echogenicity, (4) poorly defined margins, (5) posterior acoustic enhancement (indicated by the red arrow), and (6) some margins with vague angularity (indicated by the red triangle). Features (5) and (6) are more likely to be misclassified.

Compared with the unaided junior readings, the AI-assisted strategy achieved an accuracy of 0.894, precision of 0.931, recall of 0.747, specificity of 0.971, and an F1 score of 0.829, with improvements observed across all metrics (Fig. **S1**). The YOLO-v7 and YOLO-v8 models showed the potential to accurately diagnose lesions that the junior radiologist failed to successfully classify (Figs. **6C**, **7**).

## 4 DISCUSSION

### 4.1 Rationale and Innovation of the Study

Ultrasound has become an important tool for the early screening of breast cancer. According to BI-RADS classification, category 4A represents a 2% to 10% probability of malignancy. Clinicians generally recommend that lesions classified as 4A or higher should be biopsied for further diagnosis. Therefore, accurate interpretation and classification of lesions are critical in determining whether patients undergo additional invasive examinations. However, correct image interpretation is often limited by examination conditions and influenced by the radiologist’s experience, which is particularly significant in the early detection of breast cancer. In this study, two object detection models, YOLO-v7 and YOLO-v8, were trained and their performance compared in the automatic classification of breast lesions, with the aim of using deep-learning models to assist radiologists, improve the accuracy and consistency of lesion classification, and reduce dependence on experience.

### 4.2 Transfer Learning and Model Performance

Transfer learning is a method that uses a pre-trained model trained on other tasks to fine-tune a target task, reducing model training time and improving performance [26]. The YOLO pre-trained models used in this study were trained on the MS COCO dataset [27], which contains 80 categories and 118,000 images. We applied these YOLO pre-trained models for transfer learning on an internal breast ultrasound image dataset established by our center. During training, images collected by various ultrasound devices were included to increase data diversity and enhance the models’ generalization ability.

Ultimately, the YOLO-v8 model demonstrated faster convergence during training than the YOLO-v7 model. Training and validation loss curves further indicated stable convergence without noticeable oscillation, and consistent performance on the external BUSI dataset supports the robustness of the proposed models. This improvement is primarily due to differences in the network architectures of YOLO-v7 and YOLO-v8. Although YOLO-v7 achieved better performance than previous YOLO versions by extending the efficient layer aggregation network and improving model scaling techniques, the anchor-free mechanism introduced in YOLO-v8, along with enhancements to the backbone network, further improved performance.

The results show that the YOLO-v8 model has higher accuracy, recall, specificity, and F1 score than the YOLO-v7 model on the external test set, indicating that the overall diagnostic effect of the YOLO-v8 model is slightly better than that of the YOLO-v7 model. This finding is also consistent with the results of other researchers using YOLO. Shaikh *et al*. [28] used multiple versions of the YOLO model network, including YOLO-v7 and YOLO-v8, to train and test the recognition of oil palm tree images. The statistical recall, precision, and F1 score showed that the YOLO-v8 model generally better recognizes oil palm trees in images than the YOLO-v7 model. This may be related to the anchor-free mechanism and the more advanced feature-extraction architecture used by YOLO-v8. In previous versions of the YOLO model, including YOLO-v7, object detection relied on a multi-scale anchor mechanism that uses predefined anchors to help the network locate targets of different scales. The anchor-matching process may increase computational complexity and training time, especially when dealing with a large number of small objects or densely populated scenes, which can make the model's computation inflexible. The anchor-free mechanism relies on directly predicting the target center, key points, or bounding boxes, which makes it easier to adapt to different target distributions, avoids problems that may arise from anchor setting, and reduces computational cost [29]. However, the performance of the two models on the internal test set images in our study is the opposite. The YOLO-v7 model has higher mAP50, recall, precision, and F1 score on the internal test set than the YOLO-v8 model. These results show that the YOLO-v7 model exhibits some overfitting on the training set, while the YOLO-v8 model shows better generalization. This consideration relates to the following two aspects: On the one hand, the YOLO-v7 model undergoes more training epochs than the YOLO-v8 model. On the other hand, the anchor-free mechanism and the deeper network architecture also enhance the robustness of the YOLO-v8 transfer learning model.

The models' inference speed also supports practical deployment. On an RTX 4090 GPU, YOLO-v8 achieved an average per-image processing rate of approximately 250 FPS, while YOLO-v7 reached about 145 FPS. These rates are well above the 10–30 FPS typically required for real-time breast ultrasound imaging, indicating that the proposed models can operate efficiently within routine clinical workflows on modern GPU workstations.

### 4.3 Comparison with Radiologists

To further verify the diagnostic effect of the models, we compared the diagnostic results of junior and senior radiologists while testing the two deep-learning models on the external test set. Compared with the diagnostic results of the senior radiologist, the accuracy of the junior radiologist in the external test set did not differ significantly, but the recall rate was lower. The junior radiologist misdiagnosed more malignant cases as benign, possibly for many reasons. First, the junior radiologist may find distinguishing the malignant signs of lesions vaguely presented in the image difficult, leading to a high misdiagnosis rate for malignant cases. In addition, the junior radiologist may be too cautious in the diagnosis process and avoid giving too high a BI-RADS classification. Although the senior radiologist has a higher recall rate for malignant lesions in the diagnosis than the junior radiologist, the specificity and precision are relatively low. Previous studies have also shown that radiologists often show a high recall rate but insufficient specificity [30, 31]. This means that the false positive rate of the diagnosis is high, and some patients undergo unnecessary biopsies and psychological pressure. In order to consider the relationship between diagnostic recall and specificity in a more balanced way, we included the F1 score in the statistical analysis to comprehensively consider the diagnostic effect and balance the missed diagnosis rate and misdiagnosis rate. It can be seen that the F1 score of the diagnosis results of the junior radiologist is significantly lower than that of the senior radiologist, indicating that the level of senior doctors is significantly higher. In comparing the transfer learning models and the radiologists’ diagnosis, the YOLO-v7 and YOLO-v8 models showed excellent performance in the classification and edge recognition of lesions. They showed the potential to accurately and successfully diagnose lesions that junior radiologists failed to classify. Overall, the diagnostic accuracy, specificity, and precision of the YOLO-v7 and YOLO-v8 models are significantly higher than those of the senior radiologist, while the recall rate is reduced. According to the comprehensive evaluation of the F1 score, the F1 score of the YOLO-v7 model is slightly higher than that of the senior radiologist overall, but the difference is not significant; the YOLO-v8 model achieved a significantly higher F1 score than that of the senior radiologist. The AI-assisted analysis further demonstrates that YOLO-based models can function as real-time decision-support tools to enhance inter-reader consistency and reduce the overestimation of BI-RADS 4 lesions. These findings indicate that the proposed models, particularly YOLO-v8, have achieved diagnostic performance at the level of a senior radiologist and can effectively assist junior radiologists in improving diagnostic accuracy.

### 4.4 Study Limitations

Our study still has several limitations. First, the models did not incorporate patient clinical information. In real diagnostic workflows, radiologists consider medical history, physical examination findings, and other imaging modalities, such as mammography or MRI, which are critical for decision-making, especially when lesion features are subtle or images are blurred. Second, only static two-dimensional images were used for model training, which limits the ability to capture temporal features available in cine clips or multi-view sequences, such as lesion vascularity changes. Third, dataset partitioning was performed at the image level after removing highly redundant images. Although each lesion was documented across multiple scanning planes and imaging modes to capture complementary features, images from the same lesion could still appear in different subsets, potentially leading to optimistic internal performance estimates. Fourth, a fixed low confidence threshold was applied at test time to prioritize sensitivity, rather than tuning the operating point on a validation set, which may influence the ranking of models. Moreover, the current analysis was performed on a per-image basis without explicit aggregation for patients with multiple lesions, which limits direct case-level decision support. Our internal dataset also comes from a single center, and only two radiologists participated in the image interpretation. In addition, although an asymmetric augmentation strategy was used to mitigate the marked class imbalance, we did not conduct dedicated ablation analyses of different augmentation factors. Indirect evidence supports the effectiveness of this approach, but a more systematic evaluation on larger multi-center datasets is needed to provide stronger validation. The external BUSI dataset used for independent validation provides limited metadata and lacks standardized information on lesion depth, morphology, and BI-RADS category, preventing detailed stratified performance analysis. Future studies should therefore include multi-center datasets with richer lesion characteristics and acquisition parameters to enable clinically meaningful stratified evaluations and to address the inherent complexity of breast ultrasound data, including variability in breast density, patient demographics, and acquisition protocols [32]. Integration with existing PACS and ultrasound reporting systems will require additional software development and validation within clinical workflows, and successful clinical deployment will also depend on the appropriate training of sonographers and radiologists, as well as the availability of user-friendly visualization or explainability tools. Privacy-preserving strategies such as federated learning may further be required for multi-center collaborations to ensure data protection while enabling large-scale validation [33]. In parallel, future work should continue to optimize these aspects by developing multimodal deep-learning models, incorporating cine-loop or multi-view video training, refining augmentation strategies with systematic validation, and expanding validation to prospective multi-center cohorts to enhance diagnostic performance and clinical applicability.

## CONCLUSION

Both YOLO-v7 and YOLO-v8 models demonstrated diagnostic accuracy comparable to that of experienced radiologists in breast lesion classification, with YOLO-v8 showing improved generalization and training efficiency. These findings suggest that deep learning models, especially YOLO-v8, have the potential to support clinical decision-making and reduce variability in ultrasound interpretation. Future studies should focus on multi-center validation and integration of multimodal data to further improve robustness and clinical applicability.

## Figures and Tables

**Fig. (2):**
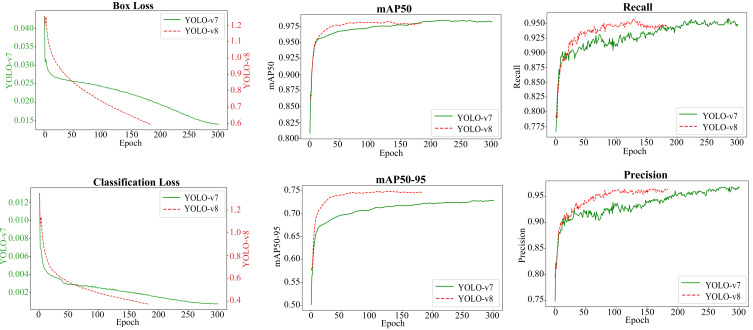
Training performance of two deep-learning models.

**Fig. (4):**
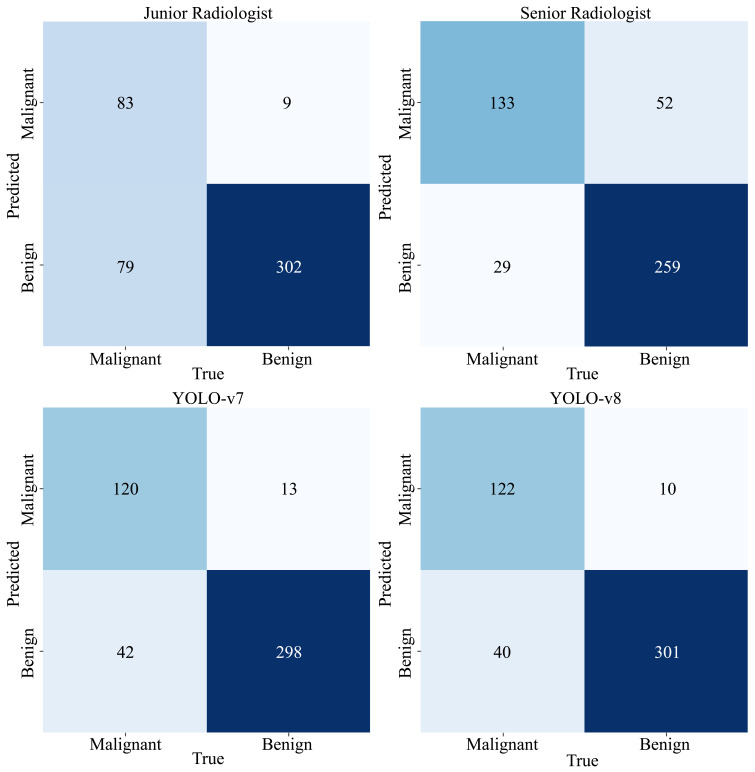
Confusion matrix of diagnostic results from models and radiologists on the external test set.

**Table 1:** Image distribution of training, validation, and internal test sets.

-	Benign	Malignant	Total	Benign-to-Malignant Ratio
Training Set	4132	1487	5619	2.78
Validation Set	522	181	703	2.88
Internal Test Set	512	191	703	2.68
Total	5166	1859	7025	2.78

**Table 2:** Image and class distribution in each dataset after data augmentation.

-	Benign	Malignant	Total	Benign-to-Malignant Ratio
Training Set	41320	29740	71060	1.39
Validation Set	5220	3620	8840	1.44
Total	46540	33360	79900	1.40

**Table 3:** Equipment used for training and related parameters.

**Name**	**Parameters**
Operating System	Windows 11, 64-bit
Processor	13th Gen Intel Core i9-13900H
Installed RAM	128 GB
GPU	NVIDIA GeForce RTX 4090
VRAM	24 GB
Programming language	Python 3.9.19
Deep Learning Framework	PyTorch 1.11.0

**Table 4:** Diagnostic results of models and radiologists on the external test set.

-	-	Accuracy	Recall	Specificity	Precision	F1 Score
Reader Study	Junior Radiologist	0.814	0.512	0.971	0.902	0.654
	Senior Radiologist	0.829	0.821	0.833	0.719	0.767
Deep Learning model	YOLO-v7	0.884	0.741	0.958	0.902	0.814
	YOLO-v8	0.894	0.753	0.968	0.924	0.830

## Data Availability

De-identified data are not publicly available due to patient privacy and institutional policy, but may be obtained from the corresponding author [H.S.] on reasonable request with approval of the Research Ethics Committee.

## LIST OF ABBREVIATIONS

IARC: The International Agency for Research on Cancer
ACR: The American College of Radiology
BI-RADS: The Breast Imaging-Reporting and Data System
CAD: Computer-aided diagnosis
YOLO: You Only Look Once
BUSI: Dataset of Breast Ultrasound Images
mAP: Mean average precision
IoU: Intersection over union
AP: Average precision
TP: True Positive
TN: True Negative
FP: False Positive
FN: False Negative
95% CI: 95% confidence interval
P-R: Precision-Recall
MRI: Magnetic resonance imaging

## STANDARDS OF REPORTING

STROBE guidelines were followed.
