# Supplementary material for: Application of YOLO-v7 and YOLO-v8 Transfer Learning Models in Breast Lesion Classification and Diagnosis
Source: Curr Med Imaging. 2026 Feb 2;22:e15734056422406. doi: 10.2174/0115734056422406251128144702 (PMC13470787; doi:10.2174/0115734056422406251128144702)
Supplement: Supplementary file 1 [file CMIM-22-E15734056422406_SD1.pdf]

1875-6603/26 *Send Orders for Reprints to reprints@benthamscience.net*

BENTHAM SCIENCE

Current Medical Imaging

Content list available at: https://benthamscience.com/journals/cmir

Supplementary Material

Application of YOLO-v7 and YOLO-v8 Transfer Learning Models in Breast Lesion Classification and Diagnosis

Kaiting Jiang[1,#, 2], Yuegui Wang[1,#], Haiquan He[1,2], Hong Chen[1] and Haolin Shen[1,*]

[1]*Department of Ultrasound, Zhangzhou Affiliated Hospital of Fujian Medical University, Zhangzhou 363005, China*
[2]*Clinical Medical College, Fujian Medical University, Fuzhou 350004, China*

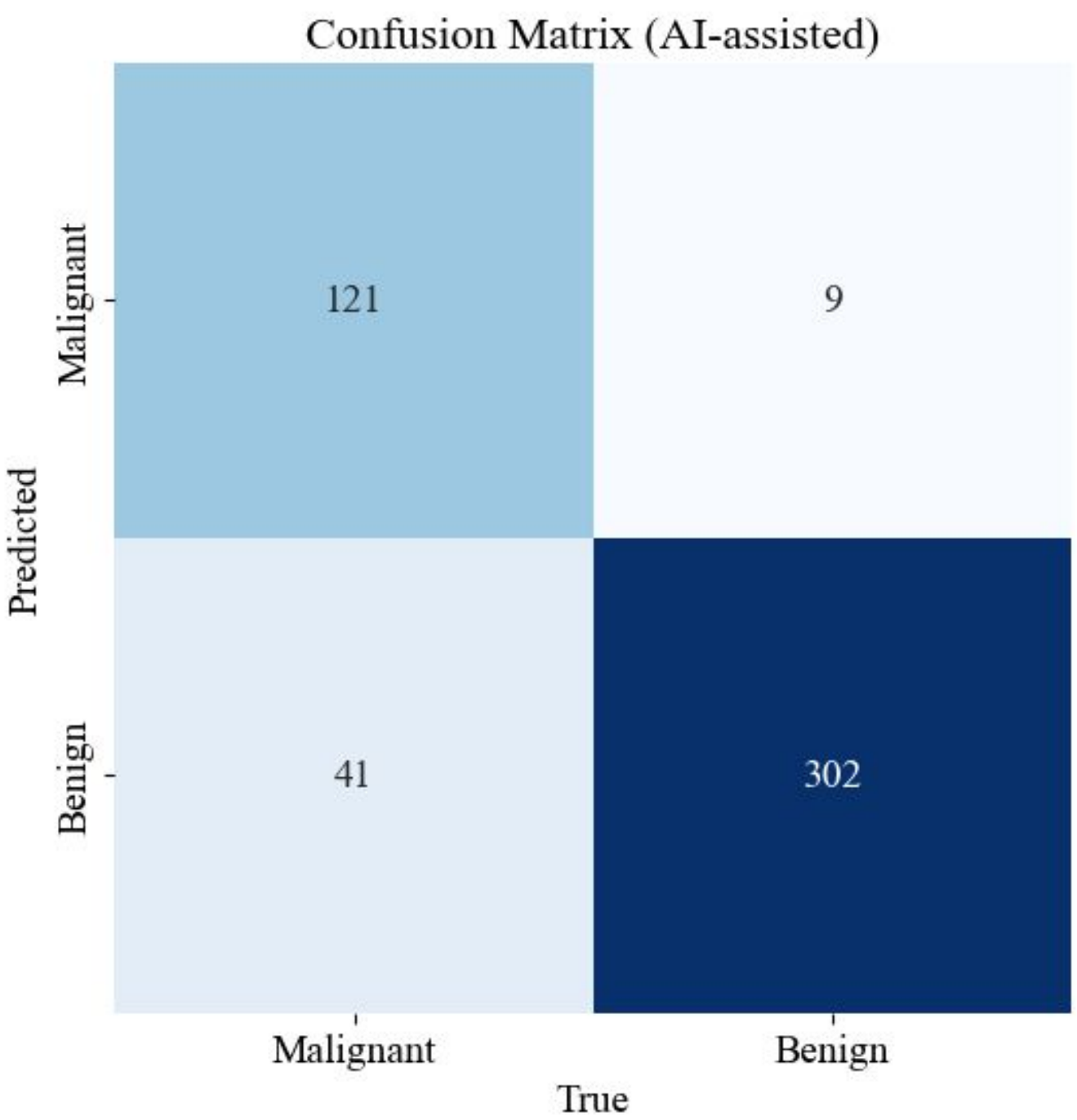

Fig. (S1). Confusion matrix showing diagnostic results for an AI-assisted junior radiologist on the external test set.

© 2026 The Author(s). Published by Bentham Science Publisher.

This is an open access article distributed under the terms of the Creative Commons Attribution 4.0 International Public License (CC-BY 4.0), a copy of which is available at: https://creativecommons.org/licenses/by/4.0/legalcode. This license permits unrestricted use, distribution, and reproduction in any medium, provided the original author and source are credited.

HOW TO CITE:
Jiang K, Wang Y, He H, Chen H, Shen H. Application of YOLO-v7 and YOLO-v8 Transfer Learning Models in Breast Lesion Classification and Diagnosis. Curr Med Imaging, 2026; 22: e15734056422406.
http://dx.doi.org/10.2174/0115734056422406251128144702
